# Artificial Intelligence Algorithms in Visual Evoked Potential-Based Brain-Computer Interfaces for Motor Rehabilitation Applications: Systematic Review and Future Directions

**DOI:** 10.3389/fnhum.2021.772837

**Published:** 2021-11-25

**Authors:** Josefina Gutierrez-Martinez, Jorge A. Mercado-Gutierrez, Blanca E. Carvajal-Gámez, Jorge L. Rosas-Trigueros, Adrian E. Contreras-Martinez

**Affiliations:** ^1^División de Investigación en Ingeniería Médica, Instituto Nacional de Rehabilitación Luis Guillermo Ibarra Ibarra, Mexico City, Mexico; ^2^Escuela Superior de Cómputo, Instituto Politécnico Nacional, Mexico City, Mexico

**Keywords:** BCI, visual stimulation, classification, performance metrics, steady state visually evoked potentials, P300, functional electrical stimulation, virtual reality

## Abstract

Brain-Computer Interface (BCI) is a technology that uses electroencephalographic (EEG) signals to control external devices, such as Functional Electrical Stimulation (FES). Visual BCI paradigms based on P300 and Steady State Visually Evoked potentials (SSVEP) have shown high potential for clinical purposes. Numerous studies have been published on P300- and SSVEP-based non-invasive BCIs, but many of them present two shortcomings: (1) they are not aimed for motor rehabilitation applications, and (2) they do not report in detail the artificial intelligence (AI) methods used for classification, or their performance metrics. To address this gap, in this paper the PRISMA (Preferred Reporting Items for Systematic Reviews and Meta-Analyses) methodology was applied to prepare a systematic literature review (SLR). Papers older than 10 years, repeated or not related to a motor rehabilitation application, were excluded. Of all the studies, 51.02% referred to theoretical analysis of classification algorithms. Of the remaining, 28.48% were for spelling, 12.73% for diverse applications (control of wheelchair or home appliances), and only 7.77% were focused on motor rehabilitation. After the inclusion and exclusion criteria were applied and quality screening was performed, 34 articles were selected. Of them, 26.47% used the P300 and 55.8% the SSVEP signal. Five applications categories were established: Rehabilitation Systems (17.64%), Virtual Reality environments (23.52%), FES (17.64%), Orthosis (29.41%), and Prosthesis (11.76%). Of all the works, only four performed tests with patients. The most reported machine learning (ML) algorithms used for classification were linear discriminant analysis (LDA) (48.64%) and support vector machine (16.21%), while only one study used a deep learning algorithm: a Convolutional Neural Network (CNN). The reported accuracy ranged from 38.02 to 100%, and the Information Transfer Rate from 1.55 to 49.25 bits per minute. While LDA is still the most used AI algorithm, CNN has shown promising results, but due to their high technical implementation requirements, many researchers do not justify its implementation as worthwile. To achieve quick and accurate online BCIs for motor rehabilitation applications, future works on SSVEP-, P300-based and hybrid BCIs should focus on optimizing the visual stimulation module and the training stage of ML and DL algorithms.

## Introduction

One of the most traditional neurorehabilitation strategies aimed at restoring motor functions lost due to various lesions of the nervous system [stroke, spinal cord injury (SCI), cerebral palsy, among others] is based on the neurofacilitation approach for proprioceptive stimulation and guidance of brain plasticity processes (Carr and Shephered, 2006; Hindle et al., 2012). These techniques involve passive stretching, contraction and relaxation of specific muscles groups in order to improve their flexibility and to stimulate the sensory function, muscle tone and recovery of movement patterns. Some key elements for motor and sensory functional recovery (Jang, 2013) are repetition of movement patterns (Zbogar et al., 2017), somatosensory stimulation (Hara, 2008) and the application of stimuli outside the motor and sensory pathways (visual, auditory, or proprioceptive) (Bach-y-Rita and Kercel, 2003; Bento et al., 2012; Takeuchi and Izumi, 2012; Galińska, 2015). These neurorehabilitation strategies make possible to re-educate neural tissue that is not completely damaged or to reactivate other areas to form new synaptic connections (Gordon, 2005).

To this end, various technologies (devices and strategies) have been developed to offer therapies that help patients to recover impaired motor functions. Brain-Computer Interface (BCI), Functional Electrical Stimulation (FES), and Neuroprostheses are devices proposed to improve motor and neurological functions (Iosa et al., 2012). The theoretical argument is that therapeutic interventions based on these neurorehabilitation technologies take advantage of the preserved neuro-muscular structures and functions, and that they can help to compensate or re-learn the functions previously performed by the damaged areas, thus improving the sensory-motor function (Iosa et al., 2012; Altaf, 2019).

### Principles of Brain-Computer Interfaces

The main objective of BCIs is to decipher the user’s intentions, registered from electrical, magnetic, thermal or chemical signals generated by the brain, and translate them into orders that are interpreted and translated by a computer into commands, in order to establish direct communication between the brain and external devices. These systems allow the user to interact with their environment, without using the peripheral nervous system or the muscular system, and when used in combination with proper motor or sensory stimuli and functional tasks, they can be used to assist, increase or help repair cognitive or sensory-motor functions. BCIs can be classified as invasive and non-invasive, according to the sensors that they use to collect brain signals, and as endogenous and exogenous, depending on if their experimental strategy requires external stimuli or not. Each type of BCI has advantages and disadvantages regarding its temporal and spatial resolution, computational cost, training requirements, and clinical application (Wolpaw et al., 2000; Birbaumer and Cohen, 2007).

Invasive BCIs have a high signal-to-noise ratio (SNR) that allows accurate pattern recognition or continuous decoding of kinematic parameters. However, this BCI approach face the risk of surgical complications and infections, short-term and long-term signal instabilities that degrade neural decoding of intent (Perge et al., 2013), and the challenge of maintaining stable chronic recordings (Meng et al., 2016). Due to their ease, non-invasive nature, high temporal resolution, portability and low cost, most BCIs use the surface electroencephalography (EEG) as the preferred method to obtain BCI control signals (Radaman and Vasilakos, 2017). To implement EEG-based BCI systems several protocols and paradigms (e.g., imagery or visual tasks) have been used to modulate the subject’s brain electrical activity (Abiri et al., 2019; Bonci et al., 2021).

Currently, several research centers are focused on studying the advantages of endogenous EEG based-BCIs to decode movement intention. To this end they use paradigms such as motor imagery to modulate sensorimotor rhythms of the EEG, which are recorded in the scalp over the sensorimotor brain area (Ramos et al., 2013; Thomas et al., 2013; Müller-Putz, 2018; Aggarwal and Chugh, 2019; Baniqued et al., 2021). Despite the advantages of endogenous BCIs based on motor related tasks (Aggarwal and Chugh, 2019), they generally need of a long training period to achieve voluntary control of the sensorimotor brain signals. Moreover, they present moderate performance for multiclass decoding (Boernama et al., 2021) and limited information transfer rate (ITR) (Choi et al., 2020). These shortcomings, combined with a relatively high inter-individual variability can limit the use of those systems outside of a controlled laboratory environment. Unlike endogenous BCIs, exogenous BCIs operate with brain signals known as event related potentials (ERPs) or steady state evoked potentials, which can be spawned by auditory, visual o somatosensorial stimuli (Wang et al., 2008). In the category of exogenous BCI paradigms the most widely used are those based on visually evoked potentials (VEPs)O VEPs are generated in response to visual stimuli, such as flashing lights presented to the subject quickly and repeatedly. These potentials can be controlled and characterized with relative ease, and their properties depend closely on the type and features of the visual stimulus (Kubler et al., 2001).

### Brain-Computer Interfaces Based on Visual Paradigms

If a visual stimulus is presented repeatedly at a fixed frequency in the 1–100 Hz range, a very stable response over time (in amplitude and phase) is elicited in the occipital area (Müller-Putz et al., 2005; Won et al., 2015). Those responses are called steady state visually evoked potentials (SSVEP) (Vialatte et al., 2010; Norcia et al., 2015). Recently, SSVEP-based BCIs have received increased attention because they can provide relatively high bit rates of up to 325 bits/min, while requiring little training (Vialatte et al., 2010; Gao et al., 2014; Nakanishi et al., 2018). In addition, SSVEPs are highly robust to artifacts produced by blinks and eye movements (Perlstein et al., 2003) and to electromyographic noise contamination.

On the other hand, exogenous ERPs can also be elicited when infrequent visual stimuli are interspersed with other more frequent or routine stimuli. In this case a positive peak called P300, is evoked at about 300 ms after the stimulus (Blankertz et al., 2011; Yeom et al., 2014), which can be recorded mainly at parietal and occipital zones over the scalp. P300 ERPs are typically elicited during an oddball target detection task, when a target or relevant stimulus is presented infrequently in a background of frequent standard stimuli. Its latency reflects processing speed or efficiency during stimulus evaluation, independent of the motor preparation time (Kutas et al., 1977). Many BCI applications based on the P300 ERP use graphical interfaces operating under the row/column paradigm, that evoke the P300 potential when the elements attended by the user are visually intensified (the target stimuli) (Philip and George, 2020). This paradigm requires the subject to focus his/her attention only in the target stimulus and not in any other stimuli (Polich, 2007; Guo et al., 2019; Riggins and Scott, 2019), which implies the ability to inhibiting attention drifts to irrelevant stimuli.

P300-based and SSVEP-based BCIs have been widely studied since they are considered robust systems with high ITR (Cheng et al., 2002; Rupp, 2014; Naeem et al., 2020) and good accuracy. In both cases the selected parameters of the stimulation pattern led to a trade-off between ITR and accuracy (Cecotti, 2011). Moreover, both BCI approaches have a high potential for clinical use, since they require few subject’s EEG data for training classification models. This makes them feasible for practical applications with short-term training (Polich, 2007; Yao et al., 2012), few recording channels and therefore lower computational cost than other BCI modalities (Müller-Putz et al., 2005; Kluge and Hartmann, 2007; McCane et al., 2015; Kundu and Ari, 2017; Nagel et al., 2017; Han et al., 2018). In this regard, it has been shown previously that technologies based on these two BCI modalities, can be transferred to be used not only in the clinical environment, but even at the patient’s home (Sellers et al., 2010).

### Artificial Intelligence Algorithms in Brain-Computer Interfaces

Traditional machine learning (ML) methods have been widely used in BCI applications, such as Artificial Neural Networks, Support Vector Machine (SVM) or Linear Discriminant Analysis (LDA). This classic ML approach require the use of namely manually designed techniques for EEG feature extraction (e.g., temporal, spectral and time-frequency methods, to name a few). The feature extraction plus ML technique approach presents the following problems: (1) it can only learn the features that researchers focus on, but ignores other potentially informative ones (Lecun et al., 2015); (2) methods performing well on certain subjects (with similar age or occupation) may not give a satisfactory performance on others, yielding a high subject-to-subject variability in EEG signals. For these reasons, different deep neural networks (DNN) have been proposed to overcome the challenges of ML techniques in BCI, allowing automatic feature extraction and classification, while achieving competitive performance on the target tasks. Hence, DNN have become an useful method to improve classification performance of BCI systems using EEG signals (Craik and Contreras, 2019) and evoked potentials (Kwak et al., 2017), with reduced computational cost and improved usability.

### Visual Brain-Computer Interface for Motor Related Applications

Currently, there is a growing interest in the application of VEP- and VERP-based BCI systems for people with disabilities. Systematic reviews have shown the potential of VEP-BCIs for motor rehabilitation purposes (Kaufmann et al., 2013; Lazarou et al., 2018). These systems allow the control of orthoses, prostheses, or FES devices to assist disabled patients during therapy (Stan et al., 2015; Zhao et al., 2016). The most common application of these BCI systems is for spellers (at least 30% of papers), but for the device control there are wheelchairs (Zhang et al., 2014, 2016; Turnip et al., 2015; Lopes et al., 2016; Waytowich and Krusienski, 2017; Yu et al., 2017; Chen et al., 2020), robots (Zhao et al., 2015; Çiğ et al., 2017; Venuto et al., 2017; Erkan and Akbaba, 2018; Yuan et al., 2018; Khadijah et al., 2019; Wang et al., 2020), and domotics tools (Venuto and Mezzina, 2018; Hossain et al., 2020; Lee T. et al., 2020).

Although several papers have been published on BCI applications based on visual paradigms, many of them do not report the performance of the Artificial Intelligence (AI) algorithms used for detection and classification of evoked potentials (P300 or SSVEP). Likewise, although numerous BCI papers are focused on studying and analyzing the performance of the classification algorithms, most of them do not report online tests with a specific application, either for communication, or for the control of motor assistive or rehabilitation technologies.

Traditionally, manually designed feature extraction techniques and machine learning algorithms have been used to detect and classify P300 and SSVEP signals within BCI systems (Bashashati et al., 2007; Lin et al., 2007). Common examples of feature extraction algorithms are spectral parameters, time-frequency representations, parametric models, cross-correlation and canonical correlation analysis (CCA), and matched filtering. Regarding ML classifiers used to detect EEG states or activity in BCI systems, examples are support vector machine (SVM), Linear Discriminant Analysis (LDA), fuzzy logic algorithms, and artificial neural networks, Unfortunately, these classification techniques can only learn from the features the designer focuses on, missing out on others that might be useful to improve their performance. Therefore, in recent years, deep learning techniques such as convolutional neural networks (CNN), recurrent neural networks (RNN), or deep belief networks (DBN) have been used in BCIs to overcome the aforementioned shortcomings of traditional ML methods (Cecotti, 2011; Cecotti and Graser, 2011; Manor and Geva, 2015; Liu et al., 2018; Shan et al., 2018).

The performance of the AI algorithms used in BCI-based spelling applications (Huang and Huang, 2017) has been evaluated through metrics such as accuracy, precision and ITR. On the one hand, BCI spellers based on SSVEP signals have reported ITR values as high as 4.5 bpm (91.04%) ITR (accuracy) (Chen et al., 2015), 325 bpm (89.83%) (Nakanishi et al., 2018) or 701 bpm (74.9%) (Nagel and Spüler, 2019). On the other hand, BCI spellers based on P300 signals have reported ITR values of 20.259 bpm (79%) (Lin et al., 2018). For hybrid spelling systems that integrate P300 and SSVEP, authors have reported an online classification accuracy of up to 93.85%, with ITR of 56.44 bpm (Yin et al., 2013). Despite the extensive number of published studies on P300-based and SSVEP-based BCI systems, only a few are focused on the rehabilitation or assistance of movements. Moreover, they generally do not report the same performance metrics used in spelling systems. Such is the case of Kaplan et al. (2016), who developed a P300-based BCI system to control phantom fingers using visual stimuli placed over them, as an “ideomotor training simulator.” On the other hand, Giménez et al. (2011) presented the electronic design of a functional electrical stimulation (FES) system and its interface with a BCI based on P300. However, these works focus on the integration of the BCI commands with the actuator, but there is a lack of information about the feature extraction methods, the AI-based classifiers, and the performance metrics they used.

### Objectives and Structure of the Paper

To address this gap, in this paper we applied the PRISMA (Preferred Reporting Items for Systematic Reviews and Meta-Analyses) methodology for a systematic literature review (SLR). The main aim of this review is to gather all relevant published works that cover the current state-of-the-art in P300 and SSVEP-based BCI systems, with an emphasis on those used for motor rehabilitation applications and the AI algorithms used for detection and classification by analyzing a large number of recent publications. It provides a general overview of the topic of interest, from traditional ML techniques to cutting-edge DL trends and underlines future challenges in the field.

The review is organized as follows: Section “Introduction” introduces key concepts and critical issues in SSVEP-based and P300-based BCI systems, and details the objectives of the review; section “Materials and Methods” describes how the systematic review was conducted, and how the studies were selected, assessed and analyzed; section “Results” focuses on presenting the papers that reported the most important performance and efficiency (accuracy and ITR) metrics of the selected studies, and describes current trends and promising approaches in this type of BCI systems. Finally, section “Discussion” discusses challenges in VEP-based BCI systems for motor rehabilitation and provides recommendations for future research.

## Materials and Methods

The SLR is based on the PRISMA methodology. To ensure data quality, we searched in the scientific databases PubMed/MEDLINE, IEEE Xplore, ScienceDirect, Scopus, Embase, and Google Scholar. The search was performed in article titles, abstracts, and keywords of works published in English language. There was no lower limit for the publication date, but the databases were searched up to June 2021. Additional records were identified through other literature sources and patent search engines like Google Patents, WIPO, and SIGA.

### Search Strategy and Selection Criteria

This SLR covers the current state-of-the-art in BCI systems based on P300 or SSVEP signals, and hybrid modalities, used in motor rehabilitation applications. In particular, the SLR is focused on the AI algorithms used for classification and the reported performance metrics in the context of the BCI applications. Three reviewers from our team carried out the search of papers to reduce the risk of selection errors and selection bias.

**FIGURE 1:**
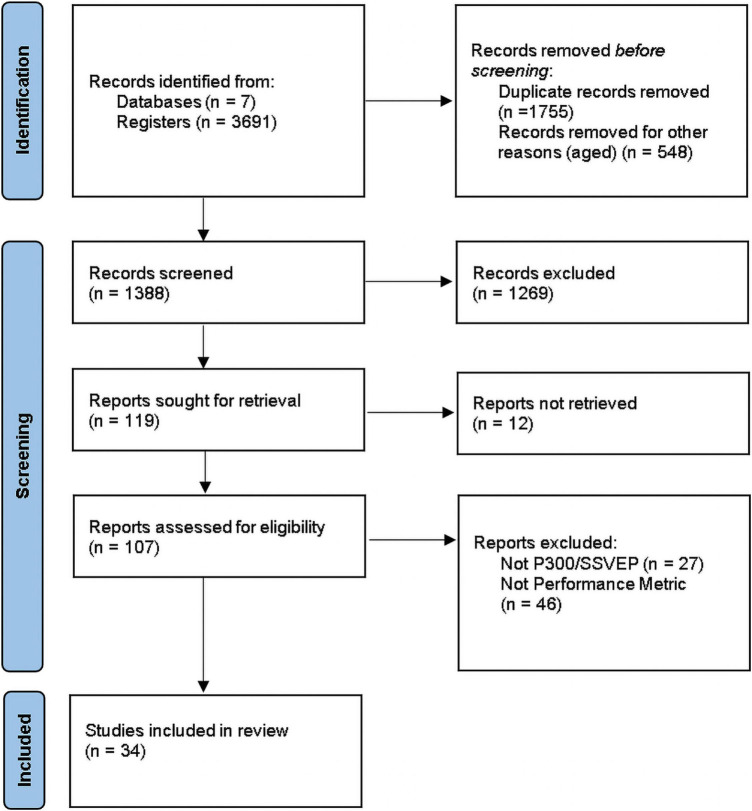
PRISMA flow diagram for the Systematic Literature Review.

The three steps involved in the manual literature search process are summarized in the PRISMA flow diagram (Page et al., 2021) in Figure 1. In the first step (Step 1- Identification) the title of articles reporting AI algorithms for SSVEP-, P300-based BCIs, as well as hybrid SSVEP/P300 BCI systems, were identified from electronic databases. Then, data extraction from abstracts and keywords was performed, and duplicate records, unrelated studies and articles published before 2011 were removed. The second step was a more detailed review of the full text articles (according to the inclusion and exclusion criteria), to assess the eligibility of the selected papers (Step 2-Screening). If the abstract did not indicate clearly whether the inclusion and exclusion criteria were met, the full text paper was also read. Papers not involving a motor rehabilitation application were removed. In the last step (Step 3-Included), the studies considered relevant and of recent advances were selected for further analysis in this SLR. The last filtering was applied to papers after reading the full text, taking into consideration whether they did not report any performance metric or did not involve a P300- or SSVEP-based BCI strategy.

### Research Questions

The goals of the SLR were translated into a set of research questions (RQ), to better explain and summarize the evidence about the AI algorithms used in P300- and SSVEP-based BCIs. In this context, the following research questions (RQs) were proposed.

RQ1: What type of evoked potential (P300 or SSVEP) is involved in the BCI’s visual paradigm?

RQ2: Is the purpose of the BCI system aimed to some motor rehabilitation application, including orthosis, prosthesis, virtual reality (VR) or FES?

RQ3: Is the classification algorithm based on AI methods?

RQ4: Are the validation methods mentioned?

RQ5: Does the paper report the performance metrics values (accuracy, ITR, etc.) of the algorithms?

RQ6: Are patients or healthy subjects involved in the study?

RQ7: What are the future challenges foreseen by the authors?

### Inclusion and Exclusion Criteria

The following medical and technical search terms were used to query the databases: “BCI,” “P300,” “SSVEP,” “brain computer interface,” “FES,” “evoked potential visual,” “neurorehabilitation,” “functional electrical stimulation.” These search terms were further combined with “artificial intelligence,” “machine learning,” “deep learning,” and “artificial neural network,” among others. Articles were also explored based on performance-related terms such as accuracy and ITR. Articles were discarded if they were not thematically relevant to the scope of this paper or they did not include tests with patients or healthy subjects. In addition to the structured literature search, a manual search of works cited in the articles included in the SLR was also conducted. Thus, some articles not identified by the original search were included in this review, if all other requirements were met. The level of evidence was not graded due to the exploratory nature of many of the studies.

### Data Extraction and Analysis

**FIGURE 2:**
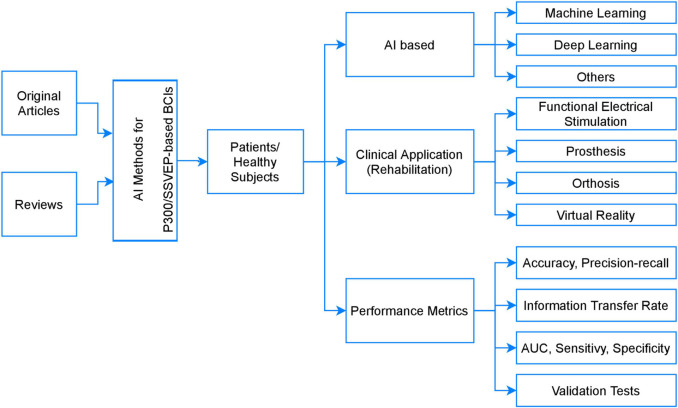
Taxonomy of the SLR: AI methods used in BCI-based P300/SSVEP systems for motor rehabilitation applications.

According to the proposed taxonomy, described in Figure 2, only two types of articles were considered: originals and reviews. The selected articles were divided into three major categories, the first one being the AI methods cluster, which provides a general overview of the used AI algorithms. The second category is a four-tiered research cluster, related to BCIs involving motor rehabilitation applications. Tier 1 contains articles involving FES systems, tier 2 provides articles related to prostheses, tier 3 considers orthoses application and tier 4 included studies aimed to the use of VR. The third category is the performance measurement cluster, which comprises the metrics employed for performance assessment of the classification algorithms.

## Results

**FIGURE 3:**
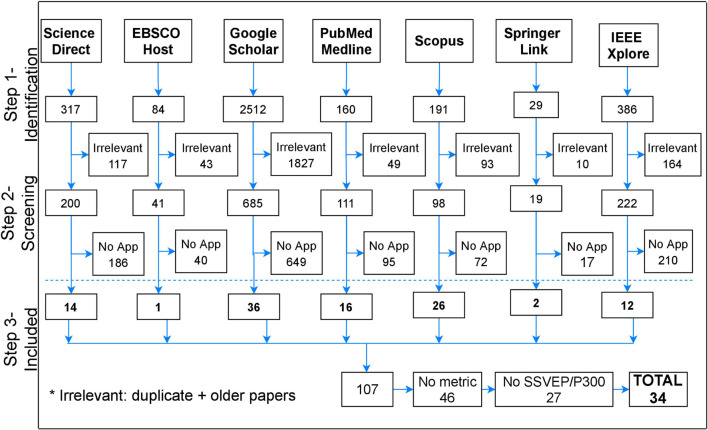
Number of records identified from each database for the Systematic Literature Review.

Three thousand six hundred and ninety one studies were retrieved from the electronic databases (Step 1-Identification), as shown in Figure 3; the first filtering step was based on the title, abstract, and keywords of the articles. After the exclusion criteria were applied, 2303 articles were discarded due to duplication or publication date prior to 2011. Of the total articles published after 2011 (1388), 1269 were excluded during full text review (Step 2-Screening) because 51.02% (702) refer to implementation and offline analysis of diverse classification algorithmic strategies, without using them in an actual application. In contrast, 28.48% (392) deal with BCI (P300- or SSVEP-based) used as speller, and 12.73% (175) for diverse applications to control wheelchairs, home appliances, robots or video games; only the remaining 7.77% (107) are focused on applying (P300- or SSVEP-based) BCIs for motor rehabilitation purposes.

The remaining 107 articles underwent a quality screening where 27 studies were eliminated, because they did not refer to either P300 or SSVEP BCIs; also 46 studies were eliminated for not specifying the performance metrics of AI algorithms. Finally, the remaining 34 papers were included as relevant to this SLR and then selected for data extraction and further analysis (Step 3- Included).

### Categorization of the Results

Table 1 shows the 34 papers considered as relevant for this SLR, of which 26.47% (9) refer to P300, 55.8% (19) to SSVEP strategy and 17.64% (6) to the hybrid BCI modality. Of the six hybrid BCIs articles, three combined P300 and SSVEP signals, and the other three combined SSVEP (2) or P300 (1) with the motor imagery paradigm. The papers were divided in five major categories, corresponding to the actuator device controlled by the P300- or SSVEP-based BCI system: FES (17.64%, *n* = 6), VR (23.52%, *n* = 8), Orthosis (29.41%, *n* = 10), Prosthesis/Exoskeleton (11.76%, *n* = 4), and RRS (Robotic Rehabilitation System) (17.64%, *n* = 6). The main application of the selected works is rehabilitation of the hand (52.94%, *n* = 18) and the lower limb (26.47%), in the latter case by means of exoskeletons and rehabilitation systems. In the VR category, objects and proprioceptive stimulation (Tidoni et al., 2017) are controlled in a virtual smart home environment (Edlinger et al., 2011).

**TABLE 1:** Artificial Intelligence Algorithms applied for detection and classification of P300 or SSVEP signals in BCI Applications for motor rehabilitation.

First author, year	BCI signal	Application/actuator	Subjects		# Electrodes	Visual stimulation pattern	Feature extraction method	Classifier	Performance		Validation Method
			Impaired	Healthy					Accuracy (%)	ITR (bpm)	
Stan et al., 2015	P300	Hand orthosis	None	9	8	Flashes: 75 ms Flash-time: 100 ms	NS	LDA	100	NS	NS
Kwak et al., 2017	SSVEP	Lower limb exoskeleton	None	7	8	5 LEDs flashing at 9, 11, 13, 15, 17 Hz with 50% DC	NR	CNN	Static: 99.28, Ambulatory: 94.93	NS	10-fold CV
Kwak et al., 2015	SSVEP	Lower limb exoskeleton	None	11	8	5 LEDs: 9, 11, 13, 15, 17 Hz with 50% DC	CCA	k-nearest neighbors	91.3	32.9	5-fold CV
Delijorge et al., 2020	P300	Robotic hand orthosis	8 ALS	18	8	2–30 random flashes	CCA	RLDA	Offline: 78.7 (target), 85.7 (non-target). Online: 89.83	18.13	5-fold CV
Zhao et al., 2016	SSVEP	FES, upper limb rehabilitation	None	5	14	Squares flashing at 12, 15, 20 Hz	Power spectrum	LDA	Offline: 79.37–85.13 Online: 54.32–87.5	Offline: 27.54	10-fold CV
Tidoni et al., 2017	SSVEP	VR, Propioceptive Stimulation	3 SCI	18	8	3 × 3 grid. flash-time: 133.33 ms dark-time: 83.34 ms	NS	LDA	83.33	1.55	NS
Yao et al., 2011	SSVEP	FES, upper limb rehabilitation	None	4	8	White blocks of lights flickering at 6.82, 7.5, 8.33, 9.37, and 12.5 Hz	5 flickering frequencies and their harmonic components	LDA	Online: 82.22	Ns	Ns
Brunner et al., 2011	Hybrid: SSVEP + P300	Moving both hand or both feet	None	12	SSVEP: 2. MI: 3.	LEDs flickering at 8 Hz (top) and LED at 13 Hz (bottom)	logarithmic band power: SSVEP and ERD	LDA	ERD: 79.9 SSVEP: 98.1 Hybrid: 96.5	ERD: 3.2. SSVEP (6.1) hybrid (6.3)	CV
Edlinger et al., 2011	Hybrid: SSVEP + P300	VR, control of virtual smart home environment	None	3	SSVEP: 8. parietal/occipital. P300: 8 frontal, central occipital, parietal	P300: rectangular matrix with characters or icons, flashed in a random order SSVEP: flickering lights (LEDs) or flickering symbols (5 -25 Hz)	SSVEP: minimum energy (ME) algorithm, P300: NA	P300: LDA, SSVEP: LDA	P300: 100	NS	NS
Su et al., 2011	Hybrid: P300 + MI	VR	None	4	P300: 14. MI: 22.	NS	P300: piecewise cubic spline interpolation+ Butterworth filter + average. MI: multiple band-pass filters	P300: SVM, MI: FLDA	Offline (MI): 92.5–100	NS	NS
Sakurada et al., 2013	SSVEP + P300	Upper limb rehabilitation,. Occupational therapy	3 (upper cervical SCI)	12	SSVEP: 3	SSVEP: 3 LEDs flickering at 8 Hz (green and blue). P300: Flash matrix	power spectrum (FFT) + CCA	SVM	Healthy: 88.46. Patients: 81.19	NS	NS
Choi et al., 2016	SSVEP + MI	FES, hand-wrist rehabilitation. SSVEP to stop FES	None	4	MI: 3 central. SSVEP: 2 occipital.	SSVEP: LED flickering at 9 Hz	MI: ERD/ERS, SSVEP: averaged Pearson’s correlation (*r*-value)	MI: FLDA SSVEP: CCA	MI: 90.485	NS	10-fold CV
Yao et al., 2012	SSVEP	FES, knee rehabilitation (movement training system)	None	2	8	a red horizontal bar, flickering light at 6.82, 8.33 and 12.5 Hz	Power spectrum	LDA	Online: 80.36–96.4	NS	10-fold CV
Duvinage et al., 2012	P300	Lower limb rehabilitation. Foot lifting orthosis	None	5	32	NS	xDAWN + two epochs average	LDA	94.30	NS	NS
Ortner et al., 2011	SSVEP	Hand Orthosis	None	7	1: O1	2 LEDS, flickering at 8 and 13 Hz	PSD	HSD	78	NS	NS
Rohani et al., 2014	P300	VR	None	5	4	NS	NS	SVM	NS	NS	NS
Son et al., 2020	SSVEP	FES, upper limb rehabilitation	None	11	19	flickering action video at 15 Hz	STFT, Power average	CSP (discriminating 2 class)	93.51	NS	10-fold CV
Chen et al., 2019	High-frequency SSVEP	Robotic arm	None	10	9: parietal or occipital	Flicker: 30, 31, 32, and 33 Hz	Spectral amplitude	FBCCA	Online: 97.75	Online: 17	NS
Li et al., 2018	SSVEP	Hand prosthesis	None	6	2: occipital	Scene graph paradigm -drinking & eating-, (8, 9.24, 10.9, and 12 Hz)	Time-frequency spectra, STFT	CCA	94.58	19.55	NS
Horki et al., 2011	SSVEP + MI	Prosthesis: artificial upper limb, elbow control	None	12	26: occipital and central	2 bars of red LEDs, flickering at 8 and 13 Hz	Sequential floating forward selection	CCA	Offline: 91	NS	10-fold CV
Koo et al., 2015	SSVEP	VR	None	3	8: central, parietal and occipital	Flickering lights at 5.5, 6.7, 7.5, and 8.6 Hz	NS	CCA for SSVEP detection	100	24.58	NS
Chu et al., 2018	SSVEP	Robotic rehabilitation system	None	6	14: frontal, parietal, occipital	Three squares flashing at 12, 15, 20 Hz	Power spectrum	LDA (voting)	82.30	27.40	NS
Gui et al., 2015	SSVEP	Lower limb rehabilitation system (hip and knee)	None	6	4: occipital and parietal	Flickering at 6.82, 7.5, 8.33, and 12.5 Hz	Spectral amplitude	LDA	92.40	NS	NS
Huang et al., 2019	P300	VR	None	6	32	3D stereo visual stimuli	NS	BLDA	96	42.51	10-fold CV
Yao et al., 2019	SSVEP	VR	None	10	9: parietal and occipital	2 stimulus presentation methods. 3D stimulus at 9, 10, 11, 12, 45 Hz	NS	FBCCA	Static mode: 92	Static mode: 22.49	Leave one-out CV
Touyama and Sakuda, 2017	Collaborative SSVEP	VR	None	8	2: parieto-occipital	two virtual cubes flickering at 6 and 8 Hz	Spectral amplitude	FLDA	95.2	NS	NS
Bhattacharyya et al., 2014	P300	Robot arm control for prosthetics application	None	5	1: Pz	Oddball-like paradigm	(Temporal) Average of 4 epochs	SVM (linear kernel)	Offline: 95.2. Online: 81.5	Online: 23.83	NS
Chen et al., 2018	SSVEP	Robotic arm control	None	12	10: P3, Pz, P4, PO3, PO4, T5, T6, O1, Oz, O2	15 targets (8–15 Hz in 0.5 Hz steps)	FBCCA for EEG decomposition	Ensemble Classifier	Robotic movement task: 92.78	49.25	NS
Casey et al., 2019	P300	Robotic arm control	None	4	6: Pz, P3, P4, PO3, PO4, and Oz	P300 speller programmed to control a robotic arm	Minimum and maximum amplitudes in the frequency domain (6 features per electrode)	2 classifiers: SVM (RBF kernel), and Random Forest	38.023	NS	NS
Achanccaray et al., 2019	P300	Robotic arm Control	None	8	16	Two images flashing randomly: a wheelchair and a robotic arm	CSP	BLDA	Training: 91.6. Test: 82.6.	NS	NS
Ding-Guo and Ying, 2012	SSVEP	FES, lower limb	None	6	NS	NS	Frequency-domain	LDA	85	NS	NS
Huang et al., 2013	P300	Elbow rehabilitation robot	None	NS	NS	Panel with 25 commands	NS	SVM	Online: 90.82	NS	NS
Okahara et al., 2018	SSVEP	Neuro-prosthesis	3-ALS	NS	1: Oz	4 × 4 LED flicker at 32–54 Hz	PSD	Classification Threshold	Online 83.3	NS	NS
Xu et al., 2021	SSVEP	Upper Limb Exoskeleton	None	5	6: O1, O2, Oz, P3, Pz, P4	4 Flickering squares at 8.57, 10, 12, 15 Hz	Frequency domain	CCA	Offline: 86.1	NS	NS

*BLDA, Bayesian linear discriminant analysis; CCA, canonical correlation analysis; CSP, common spatial patterns; DC, duty cycle; FLDA, Fisher’s Linear discriminant analysis; LDA, linear discriminant analysis; NS, non-specified; SVM, support vector machine; VR, virtual reality; CV, cross validation; FES, Functional Electrical Stimulation; MI, motor imagery; SSVEP, steady state visually evoked potentials; SCI, spinal cord injury; HSD, harmonic sum decision; STFT, short-time Fourier Transform.*

All VEP-based BCI systems were tested on healthy subjects, and only 4 (11.76%) of them included both abled-bodied participants and patients, mainly with SCI and amyotrophic lateral sclerosis (ALS). The remaining (30) works tested their systems exclusively with healthy subjects. Nine of the identified studies tested the BCI system in more than ten able-bodied subjects (Brunner et al., 2011; Horki et al., 2011; Sakurada et al., 2013; Kwak et al., 2015; Chen et al., 2018; Delijorge et al., 2020; Son et al., 2020; Zhu et al., 2020). Of the four studies that recruited both healthy subjects and patients (Sakurada et al., 2013; Tidoni et al., 2017; Okahara et al., 2018; Delijorge et al., 2020), only one (Sakurada et al., 2013) reported the classification accuracy for both patients (88.46%) and healthy subjects (81.1%). Moreover, all of them used a different number of EEG electrodes (3–8), BCI paradigms (P300, SSVEP and hybrid), and visual stimulation patterns. Also, the four studies were focused on upper limb, but they used different actuators: neuroprosthesis, orthosis, VR and rehabilitation system.

Prior to classification, some feature selection algorithm is commonly applied to (i) reduce redundancy, (ii) choose the features more related to the target mental states in the BCI, (iii) reduce the number of parameters to be optimized by the classifier, or (iv) produce faster predictions for new data. Power Spectral Density (PSD), Short-time Fourier Transform (STFT), Common Spatial Patterns (CSP), and Independent Component Analysis (ICA) are commonly used algorithms for feature extraction, but amplitude/spectral power (37.83%) and CCA (10.81%) were the most reported methods in this SLR.

Regarding the use of AI methods for classification, the most reported ML algorithms were LDA (48.64%) and SVM (16.21%), with reported accuracy range from 38.02 to 100% and ITR from 1.55 to 49.25 bpm. The best ITR (49.25 bpm) was for the SSVEP paradigm using an ensemble classifier (Chen et al., 2018). Only one study used a DL algorithm: CNN, with excellent classification accuracy (99.28 and 94.93% in static and dynamic conditions) but unspecified ITR (Kwak et al., 2017). On the other hand, only five papers reported other performance metrics besides classification accuracy: true positive rate, positive predictive value, false positive rate, Area under the ROC Curve (AUC), sensitivity and specificity. Finally, less than one out of three of the selected papers reported the validation method they used: k-fold cross-validation (29.41%, *n* = 10) and leave one-out cross validation (2.94%, *n* = 1).

### Other Results

As mentioned, hybrid VEP-based BCI systems were also found, which use two BCI control signals, each one for a specific task. For example, the hybrid SSVEP/MI system reported by Savić et al. (2012) is used to active a FES system, where the SSVEP signal is used for target selection and the MI strategy for activation of the FES-assisted reach-to-grasp of a certain object. Other hybrid BCI systems using P300 and SSVEP signals have been reported, one for controlling a smart home environment, where a SSVEP-based toggle switch was implemented to activate and deactivate the P300 BCI (Edlinger et al., 2011). Another hybrid BCI allows subjects to simultaneously imagine themselves moving both hands or both feet, while fixing the sight on one of two oscillating visual stimuli to activate an SSVEP BCI system (Brunner et al., 2011).

Regarding EEG electrodes, SSVEP and P300 BCI systems used a minimum of two recording channels for SSVEP (Li et al., 2018) and 1 for P300 (Bhattacharyya et al., 2014), and it goes up to a maximum of 19 for SSVEP (Son et al., 2020) and 32 for P300 BCIs (Duvinage et al., 2012; Huang et al., 2019). They are placed predominantly over the parietal and occipital (visual cortex) regions, in the positions P3, Pz, P4, PO3, PO4, T5, T6, O1, Oz, and O2 of the 10–20 International system for EEG electrode placement.

A key component in P300-/SSVEP-based BCI systems is the visual stimulation module. Although this element is not considered in detail in this paper, it is worth mentioning that there is a great variety of visual stimulation patterns (Amaral et al., 2017; Choi et al., 2019), ranging from flashes with variable duration (tens or hundreds of ms), with matrices of different types (LEDs, characters, or icons) to evoke P300 signals, and a range of frequencies (from 5 to 25 Hz) to produce SSVEP signals. For P300 BCIs, two strategies were used to improve the performance, 3D virtual visual stimuli (Huang et al., 2019), and overlay of smiley faces over targets (Delijorge et al., 2020).

However, if a low visual stimulation frequency is used by the visual stimulation module, the system’s ITR may be limited. To overcome this limitation, diverse stimuli colors and flickering frequencies have been proposed for hybrid BCI’s. With these variations of the visual stimulation paradigm, a good trade-off is achieved between accuracy (92.30%) and ITR (82.38 bpm), enhancing the potential to develop P300/SSVEP-based BCIs for the control of rehabilitation devices (Katyal and Singla, 2020).

## Discussion

The results of the SLR are discussed according to the Research Questions stated in section “Research Questions.”

### RQ1: What Type of Evoked Potential (P300 or Steady State Visually Evoked Potentials) Is Involved in the Brain-Computer Interface Visual Paradigm?

As shown in this SLR, despite the large number of articles related to BCI systems based on VEPs, most of them report the implementation and analysis of diverse algorithmic strategies to train and test their classification performance, without any actual application, such as motor rehabilitation. We found that using either P300 or SSVEP signals, it is possible to operate a BCI system by performing visual attention tasks. EEG signal features in those systems are extracted in the time or frequency domain, without compromising greatly the system’s accuracy and requiring little or no training.

The SSVEP signal has some advantages over the P300: (1) no mental task is required to induce the intended potential, (2) enables subjects to use the paradigm without requiring great mental load, and (3) it achieves higher ITR. However, the number of command choices in an SSVEP paradigm is generally represented by frequencies within the band of 5–20 Hz (Katyal and Singla, 2020).

SSVEP-based BCIs can encode multiple commands without any extensive user training and show potential for high-speed communication. For example, Chen et al. (2015) reported an ITR of 267 bpm in a 45-target system (Chen et al., 2015) and in Nakanishi et al. (2018) was reported an ITR of 325.33 bpm in a 40-target system. Although the efficiency and performance of different algorithms for detecting the P300 and the SSVEP in BCI applications have already been evaluated in a variety of laboratory demonstrations (Kluge and Hartmann, 2007; Kundu and Ari, 2017), many difficulties are still faced to implement this type of BCI systems for the control of devices with clinical purposes. One of these problems is the limitation in the number of available stimulation frequencies (Müller-Putz et al., 2005). One limitation of those papers is that not all of them report a full set of technical descriptions, such as the signal processing techniques for feature extraction and performance metrics of the classification algorithms, in most cases they only report classification accuracy. However, from the reported online performance of SSVEP-based BCIs (Table 1), it is clear they provide effective communication speed with good average accuracy after a very short training period (Guger et al., 2012). However, flickering lights could be disturbing for some people. In the other hand, P300-based BCIs are less accurate than SSVEP-based BCIs but are more suitable for people suffering epilepsy or people having difficulties with accurate control of the eye muscles (Allison et al., 2010).

### RQ2: Is the Purpose of the BCI System Aimed to Some Motor Rehabilitation Application, Including Orthosis, Prosthesis, Virtual Reality or Functional Electrical Stimulation?

As shown in Table 1 and Figure 2, SSVEP- and P300-based BCIs have been used in motor rehabilitation applications to drive primarily four types of actuators and then facilitate brain plasticity in patients with limb motor dysfunction. They are (1) Orthosis (Ortner et al., 2011; Duvinage et al., 2012; Stan et al., 2015; Delijorge et al., 2020) and exoskeleton (Gui et al., 2015; Kwak et al., 2015; Bhagat et al., 2016), used to perform sequences of movements to activate the hand, wrist, arm, leg or foot. (2) FES, which has been reported to be of help to regain coordination and improve performance in functional tasks (Do et al., 2011; Ding-Guo and Ying, 2012; Yao et al., 2012; McCabe et al., 2015; van Dokkum et al., 2015; Choi et al., 2016; Osuagwu et al., 2016; Zhao et al., 2016; Son et al., 2020). (3) Prosthesis (Li et al., 2018), and (4) VR (VEP-based BCI systems immersed in virtual environment) (Su et al., 2011; Koo et al., 2015; Tidoni et al., 2017; Touyama and Sakuda, 2017; Choi et al., 2019; Huang et al., 2019; Yao et al., 2019).

### RQ3: Is the Classification Algorithm Based on Artificial Intelligence Methods?

Most algorithms for classification of VEP-based BCI signals are based on AI methods. The advantages and disadvantages of each of them depend on the signal and the application. A simple and efficient ML algorithm, LDA, was among the best methods in terms of classification accuracy and ITR used in P300-based (ACC = 100% orthosis) (Stan et al., 2015) (ACC = 94.3%) (Duvinage et al., 2012), and SSVEP-based BCI systems selected in the SLR (ACC = 79%, ITR = 27.54 bpm-FES) (Zhao et al., 2016), (ACC = 83.33%, ITR = 1.55 bpm -VR) (Tidoni et al., 2017) (ACC = 82.22% -FES) (Yao et al., 2011), (ACC = 80-96% -FES) (Yao et al., 2012), (ACC = 82.30%, ITR = 27.4 bpm) (Chu et al., 2018) (ACC = 92.4%) (Gui et al., 2015), (ACC = 85% -FES) (Ding-Guo and Ying, 2012). Moreover, classification Accuracy obtained with LDA in P300-based BCI is slightly higher than with SSVEP-based BCI. Hence, LDA can be considered a first-choice ML classification algorithm for BCIs based on visual paradigms for rehabilitation applications.

Some ML classifiers such as FBCCA, FLDA, and BLDA have been proposed to improve the trade-off between accuracy and ITR of VEP-based BCI systems. They presented accuracies over 90% for both modalities (P300 and SSVEP) (Touyama and Sakuda, 2017; Achanccaray et al., 2019; Chen et al., 2019; Yao et al., 2019). The FBCCA and BLDA algorithms were superior to LDA in terms of ITR; for example, using a FBCCA (Chen et al., 2018) achieved an ACC = 92.78% with a high ITR (49.25 bpm), when an SSVEP signal was used to control a robotic arm. In the other hand, a BLDA-based classification algorithm was applied in a P300-based BCI coupled to the VR environment; in this case ACC = 96% and ITR = 42.51 bpm were achieved (Huang et al., 2019). The filter bank CCA (FBCCA) method has been extensively studied by Chen et al. (2018). This method incorporates the fundamental and harmonic frequency components to improve the detection of SSVEPs and has demonstrated its superiority over the standard CCA method (Chen et al., 2015, 2019).

The only work in the SLR that used a DL algorithm (CNN) for signal classification was (Kwak et al., 2017). In that study, the authors reported a BCI system for control of a lower limb exoskeleton via a visual stimulus generator that produced five different frequencies for SSVEP signals. They used CCA, Multivariate Synchronization Index (MSI) and CCA with k-Nearest Neighbors (CCA-KNN) to compare the classification result with three different classification methods. Using CNN-1 (three-layer network), they achieved an accuracy of up to 91.3% and an ITR of 32.9 bpm.

Beyond the works included in this SLR, DL methods have some advantages for classification of SSVEP and P300 BCI signals in comparison with the traditional ML algorithms, including:

(1) Higher Classification Accuracy (Thomas et al., 2017).
(2) DL methods reduce the dependence on manually designed feature extraction.
(3) As the size of the dataset increases, DL techniques tend to perform better than traditional classifiers (Kwak et al., 2017; Lee J. et al., 2020).
(4) The development of new powerful GPUs (graphics processing units) and cloud-based AI services have improved the cost-effectiveness of DL systems.

Despite those advantages, DL techniques have some disadvantages compared to ML algorithms:

(1) They are complex, computationally expensive, and require a large amount of data to be trained.
(2) Configuration of the different parameters of DL systems is still a major challenge.
(3) DL methods have not yet shown convincing improvements over state-of-the-art ML classification algorithms for BCI (Lotte et al., 2018).

### RQ4: Are the Validation Methods Mentioned?

Regarding validation methods, about one third of the studies reported the type of cross validation they used (1: leave one-out, 2: 5-fold, and 7: 10-fold). This data is relevant as an indicator of the robustness and confidence on the reported performance (accuracy) of the of the AI-based classification algorithms, and of their generalization ability. When the validation methods are not explicitly reported, the certainty about the results may be questionable (Abdulaal et al., 2018).

### RQ5: Does the Paper Report the Obtained Values of the Performance Metrics Values (Accuracy, Information Transfer Rate, etc.) of the Algorithms?

Two important performance criteria for classification algorithms in BCI systems are accuracy and ITR. According to BCI literature (Hwang et al., 2013), an accuracy greater that 70% must be achieved by any subject to be able to use a BCI system effectively for the control of external devices. The average classification accuracy of SSVEP-based BCI systems was 90.3% (*n* = 20), while for P300 was 85.9% (*n* = 9), and 93.41% (*n* = 6) for hybrid systems. In contrast, few works report the ITR, with a mean value of 20.88 bpm for SSVEP (*n* = 10), 28.15 bpm for P300 (*n* = 3), and 6.3 bpm for the only hybrid BCI that reported it (Brunner et al., 2011). It is worth mentioning that the average accuracy of P300 systems was lower than for SSVEP due to a single paper (Casey et al., 2019) that reported 38% classification accuracy. Without taking into consideration that article (*n* = 8) the average accuracy of P300 would be very similar to SSVEP (91.88%).

However, the above comparisons must be taken with reserve, since the number of works reporting the metrics varies a lot across modalities. Moreover, there is a high heterogeneity in different aspects of their experimental paradigms, visual stimulation features (frequencies, colors, signs, figures), subjects (healthy or patients), rehabilitation application (FES, prosthesis, orthosis, VR, etc.), length of data analysis windows, signal acquisition hardware, type (passive, active) and number of electrodes, etc. Each of those aspects affect different parts of the system that influence performance metrics, such as the complexity and execution time of the signal processing and classification algorithms.

Despite all the differences across the articles in technical and human aspects that can affect performance metrics, it is noticeable the high similarity in the average accuracy for the three BCI types considered. Regarding hybrid BCIs, they did not significantly increase the classification accuracy in comparison with single modality BCIs, as was the case for Brunner et al. (2011), with 96.5% for SSVEP and 98.1% for the MI/SSVEP hybrid modality. Moreover, most hybrid BCIs did not report the ITR value. A possible reason for this is, that in comparison with single modality VEP-BCIs, hybrid BCIs have relatively low ITRs due to more complex setups, involving one operation stage for each BCI signal, each one with a signal processing block, plus the necessary pauses between operation stages. For these and other reasons, when the users present motor imagery BCI illiteracy, single modality VEP-based BCI systems could be a better option that hybrid ones (SSVEP + MI), as suggested by Brunner et al. (2011) for SSVEP.

### RQ6: Are Patients or Healthy Subjects Involved in the Study?

All VEP-based BCI systems included abled-bodied and only a handful of them included both healthy subjects and patients with SCI or ALS disease. Several human factors directly related with the experimental setup of the BCIs, such as reaction times, mental load and fatigue, and user engagement and motivation, could have impacted the performance metrics results. Those factors become especially relevant in users with severe motor impairments. Regarding P300-based BCIs, it has been reported that the P300’s latency is higher for disabled subjects (around 500 ms) when compared to able-bodied ones (around 300 ms), and that the amplitude at the P300 peak is smaller for disabled (around 1.5 μV) than for the able-bodied subjects (around 2 μV) (Hoffmann et al., 2008). As an example, Sakurada et al. (2013) presented a hybrid (SSVEP + P300) BCI system, that compared the classification accuracy of healthy subjects (88.46%, *n* = 12) and SCI patients (81.1%, *n* = 3). These differences can be explained, at least in part, by the difficulty of patients to control eye gaze, and head or trunk posture during the BCI sessions, which could have in turn exacerbated physical and mental fatigue.

### Beyond Research Questions

Other topics of interest were identified during the development of the SLR, that fall outside the scope of the above Research Questions. These topics are discussed in the following subsections.

#### Visual Stimulation Patterns

Some studies have suggested that different visual stimuli patterns produce variations in the VEP signals, and thus have an impact on the BCI performance (Speier et al., 2017; Li et al., 2020). Mainly, low- (up to 10 Hz) and medium-frequency (13–25 Hz) stimuli have been adopted in SSVEP (Kuś et al., 2013). Although stimulation in these frequency ranges evoke SSVEPs with a large amplitude, it can be annoying or tiring for some users. A possible solution to this problem is to use high-frequency stimulation. High-frequency stimuli can decrease visual fatigue caused by flickering, thus making the SSVEP-based BCI a more comfortable system (Wang et al., 2005; Diez et al., 2011; Volosyak et al., 2011). Other visual stimulation techniques have been proposed to enhance SSVEP BCIs performance, like amplitude modulation (Chang et al., 2014), variation of the duty cycle (Shyu et al., 2013) or interpolation techniques (Andersen and Müller, 2015).

For P300-based BCIs, variations in color and arrangement of the visual stimuli (Guo et al., 2019) and overlay of targets with pictures of faces of famous people (Kaufmann et al., 2011), have shown to increase the classification performance for spelling applications. Flashing elements can change the color from blue to green at the time of intensification, (Takano et al., 2009), or 3D virtual visual stimuli can also be presented to the subject (Huang et al., 2019). However, if a low visual stimulation frequency (interstimulus interval) is used by the visual stimulation module, the system’s ITR may be limited (Mainsah et al., 2015). To overcome this limitation, diverse stimuli colors and flickering frequencies have been proposed for hybrid BCI’s achieving a good trade-off between accuracy (92.30%) and ITR (82.38 bpm) (Katyal and Singla, 2020). These approaches have the potential to enhance the development and performance of P300/SSVEP-based BCIs for the control of rehabilitation devices.

#### Electrode Setup

The configuration of electrodes (number and placement) determines the suitability of the system for daily use. In the SLR systems with 4–32 electrodes were found, predominantly located over the parietal-occipital area for SSVEP and widespread from frontal to occipital areas for P300. VEP-based BCI systems using fewer electrodes require also shorter donning times and are more user friendly than systems with many electrodes. However, if too few electrodes are used, there is a risk of not capturing all necessary features for accurate classification. This has been shown previously for both P300 (McCann et al., 2015) and SSVEP (Carvalho et al., 2015; Ravi et al., 2019) BCI systems, in studies that find optimal subsets of channels, that enhance classification accuracy. Although small subsets of electrodes (even with one or two) are selected as optimal for some users and feature extraction algorithms (McCann et al., 2015), in most cases a third or more of all available electrodes are selected through channel selection algorithms (Carvalho et al., 2015) to work properly. Interestingly, in users with low SSVEP responses (BCI illiteracy) the electrode subsets chosen through channel selection algorithms may include preferentially those located in regions (central and frontal) not typical (occipital and parietal) for this BCI modality (Carvalho et al., 2015). Likewise, it has been proposed in (visually evoked) P300-based BCIs (McCann et al., 2015) the search of non-standard sets of electrodes, to optimize the performance in individuals with motor impairments, who have little or no control of eye movements.

#### Steady State Visually Evoked Potentials and P300 Brain-Computer Interfaces for Motor Rehabilitation

Although both SSVEP and P300 BCI systems based on visual stimuli were found in this SR, there are fundamental and technical aspects of each one that can influence their suitability to be incorporated in rehabilitation applications, to name: the experimental paradigm, the degree of cognitive and sensory requirements, covert and over attention, and synchronous/asynchronous operation. These aspects are further discussed below.

First, their experimental paradigms and neurophysiological basis are essentially distinct. On the one hand, SSVEP signals directly reflects the (fixed) frequency of presentation of visual stimuli in EEG oscillations. These signals are recorded typically in occipital electrodes over the visual cortex area (Müller-Putz et al., 2005), and they reflect the sensory processing of visual stimuli. On the other hand, P300-based BCIs based on visual stimuli are designed around the oddball paradigm, in which a series of stimuli (one relevant, or target, and other irrelevant, and ignored) are presented repeatedly in random order. In this case, the key variables are the probability of occurrence of the target stimulus and the inter-stimuli interval, which can be varied randomly.

A main difference in the experimental paradigm of SSVEP and P300 BCIs is the task required for the subject while looking at the target. For the SSVEP, the only requirement is to maintain the gaze fixed on the target visual stimulus. Generally, time windows of 1–3 s are enough to identify when the subject is visually attending the target (Liu et al., 2020). In the P300 case, the user is asked to perform some mental activity for each flashing of the visual target that he or she acknowledges consciously, while ignoring the non-target stimuli. Generally, this mental task involves counting mentally the number of times that the target symbol or picture is intensified (visual stimuli) (Arvaneh et al., 2019). This is performed to engage continuously the working memory, thus involving a definite cognitive activity besides the visual attention task. Thus, cognitive (N200, P300) and visual (P100, N100) potentials are often found on EEG signals from P300 BCIs (Aloise et al., 2012). In contrast, sinusoidal-like SSVEP signals directly reflect the frequency (and harmonics) and phase of the attended stimuli (Sozer, 2018), without the need of any cognitive or behavioral task. Therefore, while P300-BCIs can be more cognitive demanding, SSVEP BCIs tend to induce more visual fatigue, especially when multiple targets are presented simultaneously (Dreyer et al., 2017). The cognitive demand of P300 BCIs may explain in part the lower average accuracy of papers included in the SLR, and why more (twice) papers used SSVEP instead of P300 signals. Moreover, of the four articles in the SLR involving patients, three were based on SSVEP and only one in P300, with relatively good levels of classification accuracy (80–90%). Therefore, differences in cognitive and visual fatigue can be also a key factor when choosing a BCI approach for patients with cognitive and motor impairment, like stroke or SCI.

One shared experimental requirement of SSVEP and P300 BCIs is that, to evoke the expected EEG activity, user attention must be focused on the current visual target for some time. For both paradigms the BCI system performs better when the sight is centered on the visual target (foveal vision) (Walter et al., 2012; Ron-Angevin et al., 2019). This is known as overt attention and is one of the key differences of SSVEP-based with P300-based BCIs, the latter having proved to work well also when visual stimuli are attended covertly, through the peripheral vision (Aloise et al., 2012). Although promising efforts have also been made to develop SSVEP BCIs based on covert attention (Zhang et al., 2010; Reichert et al., 2020), their performance still is lower than with overt attention. This aspect of visual BCIs has implication for the development of applications. In the case of motor rehabilitation of users with restrained control of gaze and neck movement (such as those with ALS or high cervical SCI), the possibility of attending stimuli covertly, and still obtaining informative EEG signals, would improve its clinical feasibility.

P300-based BCIs seem to have some advantages over SSVEP ones, since multi-target systems are feasible even using covert attention (Aloise et al., 2012), while SSVEP BCIs using this approach have been limited to a couple of targets (Zhang et al., 2010). Hence, a P300-based BCI system designed for covert attention, would allow the subject to attend visual stimuli (for selection of multiple actions or commands) while performing functional motor tasks, aided by some of the actuators mentioned in the SLR (FES, orthosis, robot, etc.). In the other hand, an SSVEP BCI system, based on overt attention, would be better suited for VR-based rehabilitation applications, with the user’s visual attention centered (overtly) in the visual target, since all stimuli and interactions are designed to be performed through the virtual environment. The papers analyzed in this SLR did not consider explicitly covert attention in their design, which remains an approach to be explored for visual BCI-based motor rehabilitation.

Another relevant aspect of visual BCI paradigms regarding their feasibility for motor rehabilitation is their type of operation: asynchronous or synchronous. In other words, if the system allows the user to convey commands at any moment (asynchronous) or only at times established by the system (synchronous) (Nooh et al., 2011). Clearly, this can be a key factor in the design of motor rehabilitation systems and interventions based on visual BCIs. For motor and neurologic rehabilitation systems and interventions, a key factor is the user’s active engagement and participation, while performing some functional tasks by their own voluntary effort or with the help of assistive technologies. This approach to rehabilitation is known as *activity-based* (Backus, 2008), and to develop systems compatible with this approach, continuous and reliable interaction between the user and the technology is highly desirable. However, these requirements are not easy to fulfill when using BCIs for the control of rehabilitation applications. Motor related BCI paradigms, such as motor imagery and motor intention, have been used extensively for BCI-controlled rehabilitation technologies (Khan et al., 2020). However, they’re limited by the number of possible commands (Lotte et al., 2010) and BCI illiteracy (Lee et al., 2019), particularly for patients with severe disability (Rupp, 2014).

SSVEP and P300-based systems have proved to obtain higher classification performance and ITR than Motor-related BCI paradigms (Rupp, 2014). Hence, the importance of developing and studying visual BCI systems for these applications or combine them with motor paradigms, like the ones found on these SLR (Horki et al., 2011; Choi et al., 2016). For P300 BCIs, multiple repetitions (5 or more) of the whole stimuli sequence are typically needed to predict accurately the user’s choice (Bianchi et al., 2021). Depending on the number of possible targets and interstimulus interval, the selection time for a single command can be relatively slow (tens of s) (Mainsah et al., 2015). Therefore, P300-based BCIs are not optimal for continuous control of actuators (Prosthesis, orthosis, FES, etc.) in the context of motor rehabilitation applications. Moreover, by its own nature, P300 BCIs operate in a synchronous way, a feature that restricts the operation of the system to certain times and cues indicated by the system. Thus, P300-based systems are often used to select and convey discrete and preprogrammed commands to the actuator, as those found in this SLR to control orthoses (Stan et al., 2015), VR systems (Rohani et al., 2014), or rehabilitation robots (Achanccaray et al., 2019). Interestingly, none of the analyzed papers combined a P300 BCI with an FES system, being an interesting possibility for future developments.

Regarding SSVEP BCIs, involving steady state signals they are suitable to implement asynchronous systems by continuously presenting the visual stimuli. In such case, the user could choose to perform a target selection task at any moment, and the system would be able to recognize it. In contrast to P300 BCIs, SSVEP BCIs have generally fewer possible targets, which correspond to the number of discernible frequencies, phases, and other features of the visual stimuli (and the evoked EEG signals). However, stimuli in SSVEP BCIs must be carefully designed since the system must be capable to identify a zero-class (non-control) besides the classes associated to the actual commands. When this is not considered, false positives are very likely to occur, like Ortner et al. who reported an SSVEP-based BCI for the control of a hand orthosis (Ortner et al., 2011). Therefore, the orthosis often opened or closed when the user did not want to convey any control signal, since the flickering lights were still within their visual field. In contrast, this would not be an issue with a P300-based BCI, that requires cognitive engagement of the subject in the task, as discussed earlier.

### Challenges and Future Directions

In this SRL, a large heterogeneity was identified in the reported BCI signals (P300, SSVEP or hybrid), applications (orthosis, prosthesis, FES, VR) and feature extraction methods, while the reported performance metrics were predominantly accuracy and ITR. Regarding classification methods, classical supervised ML algorithms (LDA and SVM) and some variations prevail, letting open the opportunity for the development of DL-based classification algorithms for visual BCI-based motor rehabilitation applications. The results of this work suggest the need to develop standard protocols for assessment of classification performance, when using VEP-based BCI systems for motor rehabilitation and assistive applications.

There are few reports of prototypes in pre-clinical stages of development with online tests. Therefore, there is a great opportunity to develop VEP-based BCI systems for motor rehabilitation. In this context, classification accuracy is a key metric to improve the BCI-user interaction and facilitate their adoption in clinical settings. Hence, strategies to improve the system’s performance for users with low accuracy must be implemented, and the visual interfaces must be closely adapted to the user needs. Special attention should be paid to the visual stimulation module since stimulus patterns have a direct impact on the performance of P300 or SSVEP-based BCIs.

Also, it is important to investigate further the application of VR combined with BCI systems where patients can be stimulated simultaneously through multiple sensory modalities: visual, auditory, and somatosensory. That way, patients can have a richer experience while playing an active role in effective rehabilitation interventions, that could potentially help to improve and accelerate the motor recovery processes. Furthermore, it is essential to carry out pre-clinical studies and controlled interventions that include patients with different conditions such as stroke, ALS or SCI. Once those studies are performed and clinical scales are evaluated, it will be possible to validate the use of these systems in the clinic.

Finally, future works should focus on optimizing the implementation and training of artificial intelligence algorithms (especially DL-based methods) to enhance classification performance and achieve faster and more efficient online P300-based and SSVEP-based BCI systems. Only then, these systems could enhance their potential for the development of rehabilitation interventions aimed to help in the recovery of lost motor functions.

## Data Availability Statement

The original contributions presented in the study are included in the article/supplementary material, further inquiries can be directed to the corresponding author.

## Author Contributions

JG-M conceived and planned the SLR methodology, performed the data collection and filtering, analyzed the manuscript, and wrote the manuscript with input from the other authors. JM-G contributed to the conception and planning of the work, performed the analysis of the literature, contributed to the manuscript writing, discussed the results, and commented on the manuscript. BC-G helped in the conception of the work and to the manuscript writing, discussed the results, and commented on the manuscript. JR-T participated in the manuscript writing, discussed the results, and commented on the manuscript. AC-M aided in the analysis and filtering of the literature and commented on the results and the manuscript. All authors contributed to the article and approved the submitted version.

## Conflict of Interest

The authors declare that the research was conducted in the absence of any commercial or financial relationships that could be construed as a potential conflict of interest.

## Publisher’s Note

All claims expressed in this article are solely those of the authors and do not necessarily represent those of their affiliated organizations, or those of the publisher, the editors and the reviewers. Any product that may be evaluated in this article, or claim that may be made by its manufacturer, is not guaranteed or endorsed by the publisher.
